# Construction and Validation of a Nomogram Predicting Depression Risk in Patients with Acute Coronary Syndrome Undergoing Coronary Stenting: A Prospective Cohort Study

**DOI:** 10.3390/jcdd10090385

**Published:** 2023-09-06

**Authors:** Xing Miao, Yongli Chen, Xiaoxia Qiu, Rehua Wang

**Affiliations:** 1 Department of Cardiology, Fujian Provincial Hospital, Fuzhou 350001, China; miaoxing126@126.com; 2 Department of Cardiology, Shengli Clinical Medical College of Fujian Medical University, Fuzhou 350001, China; 3 South Branch of Cardiology Department, Fujian Provincial Hospital, Fuzhou 350028, China; cyl2014@fjmu.edu.cn; 4 Department of Cardiology, Shengli Clinical Medical College of Fujian Medical University, Fuzhou 350001, China

**Keywords:** ACS, after stent implantation, depression, predictive nomogram

## Abstract

Purpose: To construct and validate a nomogram for predicting depression after acute coronary stent implantation for risk assessment. Methods: This study included 150 patients with acute coronary syndrome (ACS) who underwent stent implantation. Univariate analysis was performed to identify the predictors of postoperative depression among the 24 factors. Subsequently, multivariate logistic regression was performed to incorporate the significant predictors into the prediction model. The model was developed using the “rms” software package in R software, and internal validation was performed using the bootstrap method. Results: Of the 150 patients, 82 developed depressive symptoms after coronary stent implantation, resulting in an incidence of depression of 54.7%. Univariate analysis showed that sleep duration ≥7 h, baseline GAD-7 score, baseline PHQ-9 score, and postoperative GAD-7 score were associated with the occurrence of depression after stenting in ACS patients (all *p* < 0.05). Multivariate logistic regression analysis revealed that major life events in the past year (OR = 2.783,95%CI: 1.121–6.907, *p* = 0.027), GAD-7 score after operation (OR = 1.165, 95% CI: 1.275–2.097, *p* = 0.000), and baseline PHQ-9 score (OR = 3.221, 95%CI: 2.065–5.023, *p* = 0.000) were significant independent risk factors for ACS patients after stent implantation. Based on these results, a predictive nomogram was constructed. The model demonstrated good prediction ability, with an AUC of 0.857 (95% CI = 0.799–0.916). The correction curve showed a good correlation between the predicted results and the actual results (Brier score = 0.15). The decision curve analysis and prediction model curve had clinical practical value in the threshold probability range of 7 to 94%. Conclusions: This nomogram can help to predict the incidence of depression and has good clinical application value. This trial is registered with ChiCTR2300071408.

## 1. Introduction

Myocardial infarction (MI) is a leading cause of mortality worldwide, and percutaneous coronary intervention (PCI) is considered the most effective treatment for this condition. However, studies have reported a high prevalence of psychological problems among patients with acute coronary syndrome (ACS), particularly in those who receive percutaneous coronary intervention (PCI) [1]. These psychological problems include depression, anxiety, and other related disorders. A structured interview identified depressive disorder in an average of 20% of patients after acute myocardial infarction (AMI) [2]. Furthermore, the mental health status of ACS patients following stent implantation can impact treatment outcomes and lead to adverse effects. Prospective studies, systematic reviews, and meta-studies have consistently demonstrated significantly positive correlations between depression and both morbidity and mortality rates among ACS patients [3,4,5,6]. This correlation has been found to be independent of traditional cardiovascular disease risk factors [7,8,9,10]. Therefore, it is essential to focus on predicting and evaluating the mental health of ACS patients after stent implantation, as these efforts have become a crucial aspect of postoperative care for myocardial infarction that are helpful in promoting a good prognosis.

In recent years, the influence of life stress events within 1 year of coronary heart disease has received increasing attention. Based on previous studies, 53–69% of ACS patients experience life event stress within 1 year before the onset of the disease [11,12,13], and life events, as stressors, can induce psychological stress and increase the risk of coronary heart disease [11,14]. In addition, the risk of cardiovascular disease increases by 15% for each additional life event [11]. However, in clinical practice, medical staff often ignore patients’ attention to life stress events. At present, the history of life stress events has not been used to predict the occurrence of clinical event risk.

Depression in ACS patients after PCI is regarded as a psychological stress-stimulating reaction that is the result of excessive psychological pressure and depression [15]. In the diagnosis and treatment of this population, mood and depression are often ignored and untreated [16,17,18]. Mild depression is more common. This is related to the mainstream Psychiatric Diagnostic and Statistical Manual, China’s criteria for classifying and diagnosing mental disorders. If the early depression symptoms of ACS patients after stenting do not meet the diagnostic criteria, these patients cannot be effectively screened with mainstream diagnostic tools. As a visual tool for disease prevention [19,20], a nomogram can predict risk probability and help medical personnel make early accurate predictions and assist in the evaluations of patients.

Chen Hongyu et al. [21] reported a predictive model of depression in patients with coronary heart disease after PCI. However, this study did not verify the degree of calibration and repeatability of the model. Worldwide, there have been very few previous studies yielding models that incorporate past medical history, demographics, clinical features, stressful life events, and psychological factors in order to develop a model to assess the risk of depression after PCI.

Based on the above, this study established a nomogram for predicting depression risk after acute coronary stent implantation that is internally validated and calibrated. The specific objective of this study is to include demographic characteristics, clinical characteristics, psychological factors, and major life events in the past year in the model, which provides a strong basis for early screening and early intervention of depression risk in ACS patients.

## 2. Data and Methods

### 2.1. Patient Information

This cohort study included the clinical data of 150 ACS patients who underwent stent implantation in Fujian Provincial hospitals from 1 September 2019 to 1 September 2021. The inclusion criteria were as follows: (1) successful coronary stent implantation; (2) age between 30–80 years old; and (3) preoperative PHQ-9 score ≤4. All participants or their legal guardians provided informed consent. The exclusion criteria were as follows: (1) previous malignant tumor or severe cognitive impairment; (2) a history of mental illness (schizophrenia or schizoaffective disorder (ICD-10: F20.x or F25.x) or bipolar disorder (ICD-10: F31.x); (3) psychopharmacological drug use within 4 weeks; (4) renal function evaluation with the CKD-EPI equation estimated glomerular filtration rate (eGFR), in patients with renal insufficiency indicating renal insufficiency was defined as eGFR < 60 mL/min/1.73 m^2^; or (5) use of uric acid-lowering drugs within 1 month.

### 2.2. Methods

#### 2.2.1. Research Tools

The authors reviewed recent systematic literature reviews and empirical studies [3,5,6,7] on risk factors in ACS patients after PCI with depression. The list of risk factors included both established risk factors such as age and education level, as well as potential new risk factors such as history of chest pain, stroke, coronary stent or pacemaker implantation, coronary artery bypass grafting (CABG), smoking status, method of hospital admission, sex, sleep duration, occurrence of major life events in the past year, number of implanted coronary stents, and contrast agent allergy. The personnel involved in data collection and analysis were blinded to the study group assignments. There are the Definition of Variables in this Study, as Shown in Table 1.

##### Life Events Scale (LES)

The Life Events Scale (LES) was developed by Zhang Mingyuan et al. [22], with reference to the research of Holmes, Dohrenwend, and Yang Desen, among others. The purpose of this scale is to quantitatively assess acute life events. The LES comprises 65 different types of life events, covering various categories such as work, family, finances, and interpersonal relationships. Furthermore, this scale assigns a corresponding Life Event Unit to each event based on domestic standards, enabling quantitative research into the impact of different life events on an individual’s stress level. It is important to note that this scale is applicable to individuals aged 16 and above in China, with a stipulation that the assessment of research subjects should consider a time frame not exceeding one year [23]. In this study, we designed 11 significant life events based on the LES scale, all of which occurred within one year. These events include widowhood, divorce, remarriage, separation of couples for over a year, the death of direct relatives, job promotion, unemployment, job changes, retirement, having a second child, and the marriage of a daughter or son. We assessed these life events in our research subjects upon admission.

##### PHQ-9 (Patient Health Questionnaire-9) and GAD-7 (Generalized Anxiety Disorder-7)

The items included in the Patient Health Questionnaire-9 (PHQ-9) and Generalized Anxiety Disorder-7 (GAD-7) are based on the diagnostic criteria outlined in the DSM-IV for depression and anxiety [24,25]. These scales are widely used for assessing depressive and anxiety symptoms and have demonstrated high sensitivity, particularly when compared to other depression scales such as the Hospital Anxiety and Depression Scale (HADS), the Kessler-6 Depression Scale, the PROMIS Emotional Distress-Depression Short Form 8a (PROMIS Depression), and another PHQ-9 [26], all of which have a sensitivity of 95%. The PHQ-9 is a self-report questionnaire used to evaluate the severity of depression in individuals. It comprises nine questions that assess various depressive symptoms. The GAD-7 is a self-report questionnaire designed to gauge the severity of generalized anxiety disorder (GAD) in individuals. It consists of seven questions that examine different anxiety symptoms. Each question is scored on a scale from 0 to 3, with 0 indicating “Not at all” and 3 indicating “Nearly every day”. The total score on the PHQ-9 can range from 0 to 27. The total score on the GAD-7 can vary from 0 to 21. Both questionnaires have five severity levels: “none” (total score of 0–4), “mild” (total score of 5–9), “moderate” (PHQ-9: total score of 10–14; GAD-7: total score of 10–13), “severe” (PHQ-9: total score of 15–19; GAD-7: total score of 14–18), and “very severe” (PHQ-9: total score of 20–27; GAD-7: total score of 19–21).

In this study, the PHQ-9 was used to assess depression by asking participants if they had experienced a series of depression symptoms in the past two weeks in order to screen for depression symptoms. Both the PHQ-9 and GAD-7 scores were analyzed in all 150 patients upon admission and again 48–72 h after surgery to monitor changes in symptoms.

#### 2.2.2. Data Collection Methods

The study was conducted in two stages: predictor data collection and diagnosis of depressive state. Demographic and clinical characteristic data, and history of major life events in past year, including body mass index and smoking status, were extracted from electronic medical records. The baseline PHQ-9 and GAD-7 questionnaires were completed by participants within 24 h of admission, and two cardiovascular attending physicians conducted the evaluation. Two to three days after the PCI surgery, two supervising nurses with relevant psychological work experience assessed participants’ anxiety and depression status using the PHQ-9 and GAD-7 questionnaires. All assessors received systematic training and were determined to be qualified before beginning their work. The questionnaires were explained using a unified guide, and a liaison officer was responsible for contacting participants and arranging face-to-face or online follow-ups at a mutually agreed-upon time.

#### 2.2.3. Statistical Methods

The data were analyzed using SPSS 22.0 and R 3.4.2. Categorical variables are presented as *n* (%), and continuous variables are presented as the mean (SD) or median (interquartile range). The normality of data was assessed using the Kolmogorov–Smirnov test. Independent-sample *t* tests or Mann–Whitney U tests were used to compare continuous variables between groups, while chi-squared tests were used for categorical variables. Significant predictors with *p* < 0.05 in univariate analysis were included in multivariate logistic regression. Odds ratios (ORs) and 95% confidence intervals (CIs) were used to estimate the strength of the association between depression risk after PCI and predictive factors. The “forward: LR” method was used for variable selection, and a column chart was constructed to visualize the results. The discriminative ability, predictive accuracy, and clinical application value of the model were evaluated using ROC curves, calibration plots, and decision curve analysis. Graph Pad Prism 8.0 was used to plot the ROC curve, while R3.4.2 was used to generate the calibration plot and decision curve analysis. Statistical significance was set at a two-sided *p* value of less than 0.05.

### 2.3. Ethical Considerations

This study has received approval from the Ethics Committee of Fujian Provincial Hospital (Ethics number: K2020-08-009). All participants have been assured that their involvement is entirely voluntary, and that their information will be kept confidential. This study has been registered in the Chinese Clinical Trial Registry, and the trial is registered with ChiCTR2300071408.

## 3. Results

A total of 695 participants were identified from the Department of Cardiology, Fujian Provincal Hospital. Of these, 445 individuals were excluded and 256 did not meet eligible criteria; 124 met exclusion criteria, and 65 refused to participate. As a result, 150 eligible patients were recruited.

### 3.1. Incidence and Comparison of Depression in ACS Patients after PCI

In the present study, 150 participants with a mean age of 65.4 ± 10.3 years were included, and all were effectively followed up. Among them, 82 (54.7%) developed depression after PCI. Comparisons between patients with and without depression in terms of different characteristics were conducted, as shown in Table 2.

### 3.2. Logistic Binary Regression Analysis of Risk Factors for Depression in ACS Patients after PCI

In the univariate analysis, variables with *p* values > 0.05 were excluded from the “forward: LR” logistic regression model. Three predictor variables, namely, the occurrence of major life events, postoperative GAD-7 scores, and baseline PHQ-9 scores, with *p* values < 0.15, were included in the multivariate logistic regression analysis, as shown in Table 3.

### 3.3. Establishment of a Nomogram for Predicting Depression Risk in ACS Patients after PCI

**Figure 1:**
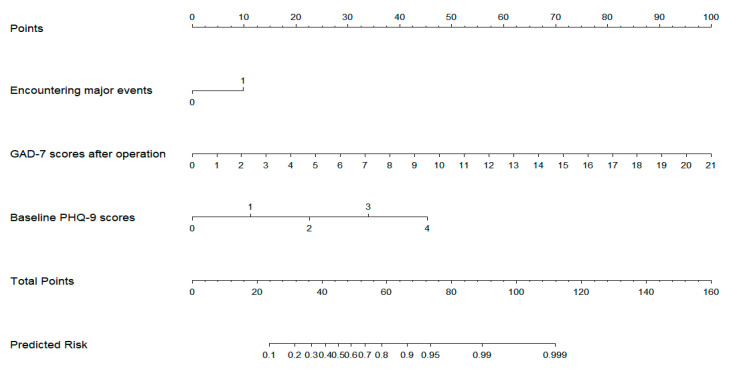
Nomogram for predicting the probability of depression after coronary stent implantation.

A column chart prediction model for predicting the risk of depression in ACS patients after PCI was constructed using logistic regression analysis, as shown in Figure 1. The model assigns scores from 0 to 100 to each variable factor, and the scores of all factors are added to obtain the total score of the model. Based on the total, the risk of early depression in ACS patients after PCI, which corresponds to the risk probability, can be predicted. The risk of depression after PCI increases with an increase in the total score. For the “History of major life events” variable, values were 0 = No and 1 = Yes. For example, consider a patient who has experienced a major life event in the past year, a post-stenting GAD-7 scale score of 7, and a baseline PHQ-9 score of 2. The patient’s corresponding scores on the column chart were 10, 33, and 22 points, respectively, with a total score of 65 points, and the corresponding risk probability of depression score was 0.89.

### 3.4. Validating and Calibration a Nomogram Predicting Depression Risk in ACS Patients after PCI

**Figure 2:**
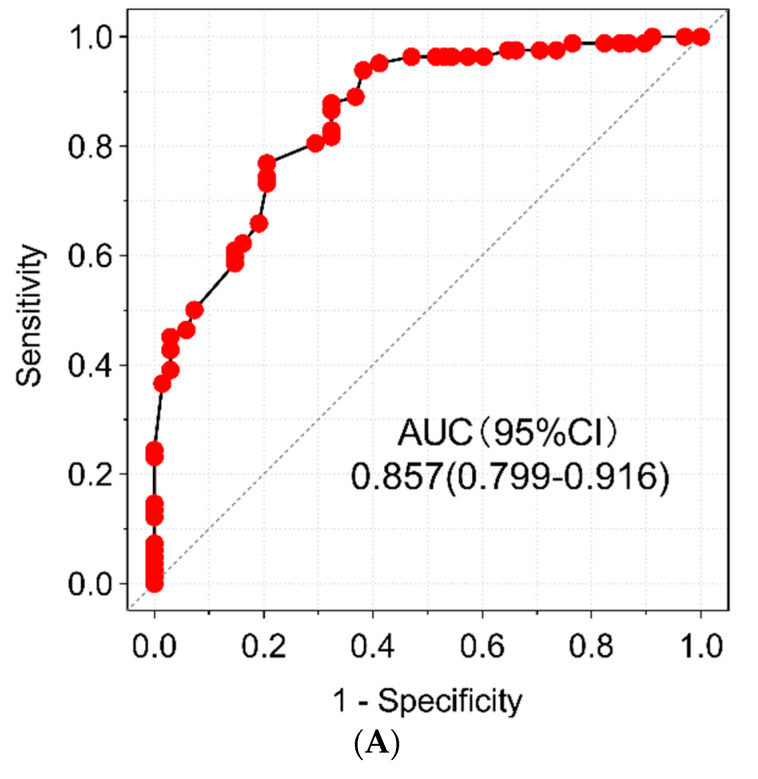

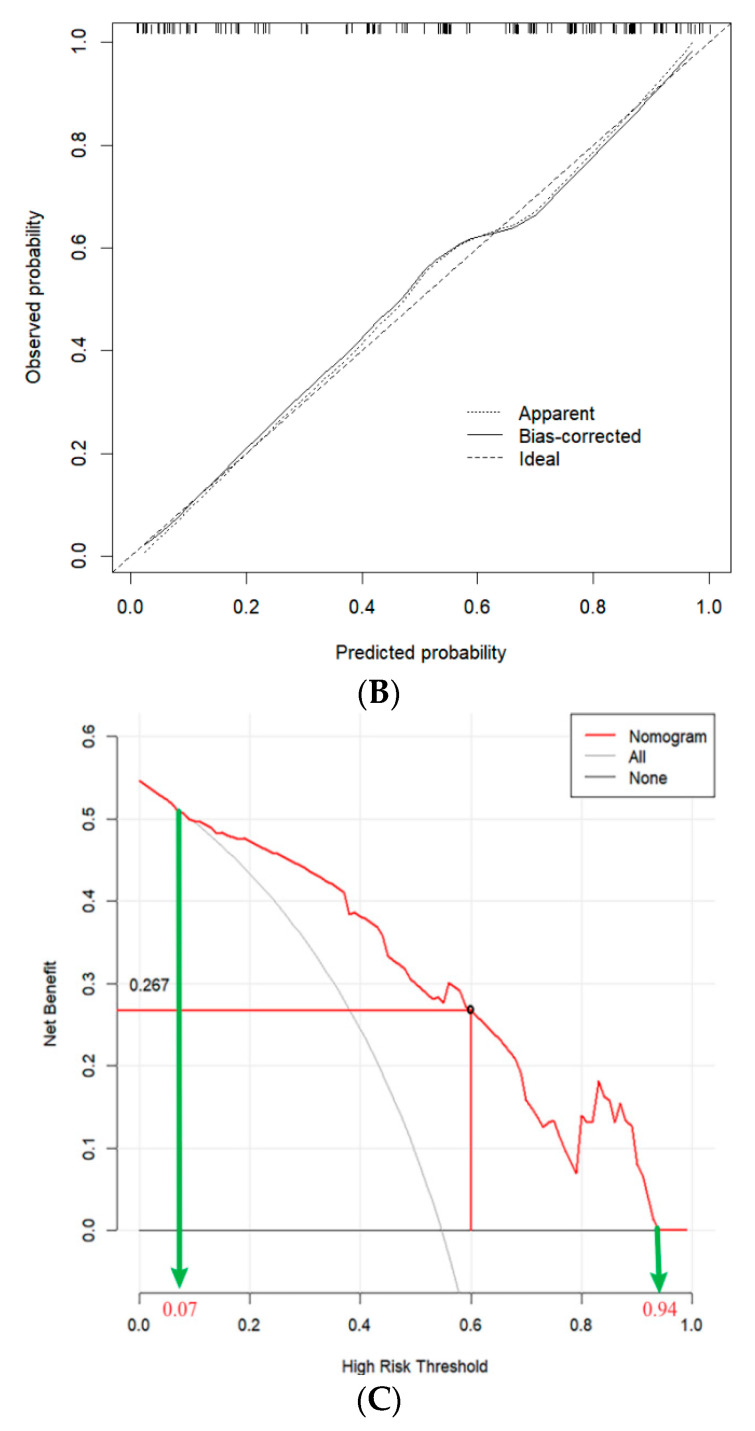
(**A**) ROC curve of the predictive nomogram for depression risk in ACS patients after PCI. The red dots are mean the sensitivity and specificity generated by the difference with the decision threshold value. (**B**) Calibration chart of the nomogram predicting depression risk in ACS patients after PCI. (**C**) Decision curve analysis of a nomogram predicting depression risk in ACS patients after PCI.

The depression risk scoring model for ACS patients after PCI showed good discriminative ability, with an AUC of 0.857 (95% CI = 0.799–0.916), as shown in Figure 2A. Internal validation using 1000 bootstrap resamples also yielded a good AUC of 0.849. The nomogram calibration plot, as shown in Figure 2B, showed acceptable calibration, with a Brier score of 0.15, indicating that the predicted probabilities of the calibration plot were well aligned with the clinical outcomes. Decision curve analysis (DCA), as shown in Figure 2C, indicated that high-risk patients with depression after coronary stent implantation can receive greater net benefits than the “treat all” or “no treatment” strategies when the threshold probability falls between 7% and 94%. Assuming a diagnostic and treatment threshold probability of 60%, among every 1000 people who use this model, 267 people will benefit from it without harming the interests of others. These findings suggest that the model has potential clinical value.

## 4. Discussion

### 4.1. The Incidence of Depression Is High in ACS Patients after PCI

Studies have demonstrated that among patients with acute ST-segment elevation myocardial infarction (STEMI), the highest rates of anxiety and depression occur within 24 h after PCI, the lowest rates occur at discharge, and the rates gradually increase within one year after discharge [25]. In this study, the results indicated that 68% (102/150) of ACS patients experienced stress related to major life events in the past year, 54.7% (82/150) of ACS patients experienced depression within 72 h after PCI, and 40% (60/150) of ACS patients experienced both stress related to major life events and depression. Compared to the rates in previous studies, such as those by Zhou Hongdan et al. [26] and Feng Yejiao et al. [27], the rate of depression in this study may be higher due to the use of a threshold of PHQ-9 ≥ 5 to define depression. Research has suggested that high morbidity and mortality are important stressors that contribute to early depression in ACS patients after PCI [28,29]. Additionally, physiological factors such as the inflammatory response, major nerve dysfunction, and platelet activation can also induce depression in patients after PCI [30]. Studies have indicated that individuals who experience life events are more vulnerable to depression [31], and depression is positively associated with the severity of life events [32]. Feng Yejiao et al. [27]. confirmed that the occurrence and severity of life events and depression in ACS patients were closely linked to plaque stability. Early identification of depression, particularly mild depression, in ACS patients after PCI depression, as well as attention to their life events, can be crucial in promoting their recovery and rehabilitation, reducing mortality, and decreasing the recurrence rate.

### 4.2. Analyzing the Risk Factors for Depression in ACS Patients after PCI

Logistic regression analysis revealed that life stress events in the past year, postoperative GAD-7 score, and baseline PHQ-9 were predictors of depression in ACS patients after PCI. Life event stress refers to an individual’s psychological stress response caused by changing situations or events in life [33]. If the stress level exceeds the psychological tolerance of individuals, it can easily lead to emotional disorders [34], affecting the physical and mental health of individuals. Jia Junjie et al. [35] conducted a study on life event-related stress and mental health risk in 2200 elderly people over 60 years old and found that family-related life events and negative life events had the strongest correlation with the mental health of elderly individuals. Studies have shown that ACS patients experience more acute life events before the onset of ACS than patients with stable angina pectoris [36,37]. This is related to the fact that life stress events stimulate the body’s psychological stress, leading to autonomic nervous dysfunction, accelerating the inflammatory response, and depression. Early identification and accurate intervention of depression in ACS patients after PCI, especially mild depression, is crucial for promoting the prognosis and rehabilitation of patients and reducing mortality and recurrence rates.

Recently, the GAD-7 and PHQ-9 have been recommended by the World Health Organization for the diagnosis and evaluation of anxiety and depression in general hospitals and primary care settings worldwide. These scales are known for their simplicity, as well as their reliable sensitivity and specificity for detecting anxiety and depression [22,23,38,39]. Similarly, the GAD-7 demonstrates robust operating characteristics for the detection of generalized anxiety, panic, social anxiety, and post-traumatic stress disorder [39]. A recent cross-sectional study involving 289 Japanese middle-aged and elderly women revealed that factors associated with depression included life satisfaction, social engagement, and anxiety scores [40]. These findings align with our own results, indicating that higher GAD-7 scores post-operation are associated with a greater likelihood of experiencing depression.

### 4.3. Establishing a Nomogram Predicting Depression Risk in ACS Patients after PCI

Currently, both domestic and international research endeavors have increasingly turned their attention towards predicting and addressing the risk of depression in patients with cardiovascular disease. The nomogram developed in this study serves as a useful tool for predicting the risk of depression in ACS patients after PCI. This prediction model exhibits strong predictive capabilities, excellent calibration, and valuable clinical utility. It combines multiple predictors and presents them in a visual format, allowing medical professionals to easily assess the likelihood of depression based on the sum of relevant risk factors [41]. It is not just a tool but represents a multidimensional, comprehensive assessment approach with the potential to have a profound impact on the diagnosis, intervention, and management of depression risk. Following a brief training, clinical staff can accurately predict depression incidence, pinpoint high-risk groups, tailor personalized diagnostic and treatment plans, and implement timely interventions based on the cumulative risk factors assessed using the nomogram for patients after stent implantation. Simultaneously, patients can access additional resources and information through the nomogram prediction model, enhancing their awareness of their mental health status and actively engaging in the treatment and management process. Furthermore, our nomogram model holds the potential for further research and quality improvement. Through data analysis, clinicians can gain insights into the risk factors associated with post-stenting depression, allowing for the development of more targeted measures. Overall, this nomogram model developed in this study is a significant clinical tool, it can help medical staff accurately evaluate the risk of depression in ACS patients and provide timely interventions to reduce the incidence or severity of depression, thus improving patient outcomes.

## 5. Conclusions

Studies have indicated that there are several risk factors associated with depression after PCI, including age, sex, type D personality, residence, smoking history, education level, hypertension, ACS severity, blood glucose level, NYHA classification, and health education prior to PCI [21,42]. However, this study did not develop predictive models related to these risk factors. It’s important to note that existing predictive models for depression after coronary stent implantation have certain limitations, it did not verify the degree of calibration and repeatability of the model [21]. Additionally, this study was limited by its small sample size and single-center design. Further large-scale, multicenter and prospective clinical trials are necessary in the future. High-quality randomized controlled trials are needed to explore whether depressive symptoms can be improved after controlling for risk factors.

## Figures and Tables

**Table 1:** The definition of variables in this study.

Variables	Definition
The second operation	Coronary stenting is staged due to complex coronary lesions, poor renal function that cannot tolerate contrast medium for long time, serious intraoperative complications, and in emergency PCI conditions only the culprit vessel is treated.
Take urate	Take urate-lowering drugs (such as allopurinol, febuxostat, benzbromarone) within one month.
Sleep duration ≥ 7 h/d	Sleep duration was defined by asking whether the respondents obtain ≥7 h for average of sleep per night in the last one month.
Body mass index (BMI)	Asian-Pacific BMI classification: underweight (BMI < 18.5), normal (18.5 ≥ BMI < 23), overweight (23 ≥ BMI < 25), obese (BMI ≥ 25)
Educational level	Participants with high school or less education = 1, above = 0. More than one stent = 1, one stent = 0.
Number of stents implanted Operation duration	If the operation is performed in stages, the operation duration is defined as the sum of the stages.
Encountering major events	Encountering major events referred to one of the following events occurs within one year: widowed, divorced, remarried, couples separated for more than one year, death of an immediate family member, promotion, unemployment, job change, retirement, birth of a second child, marriage of a daughter or a son.

**Table 2:** Comparison of different characteristics between patients with and without depression.

Variable	Without Depression (*n* = 68)	With Depression (*n* = 82)	*t*/*Z*/*x^2^*	*p*
Chest pain			0.087	0.769
No	16 (43.24)	21 (56.76)		
Yes	52 (46.02)	61 (53.98)		
Coronary stent implantation			1.447	0.229
No	40 (41.67)	56 (58.33)		
Yes	28 (51.85)	26 (48.15)		
Stroke *				0.378
No	67 (46.21)	78 (53.79)		
Yes	1 (20.00)	4 (80.00)		
CABG *				0.627
No	67 (45.89)	79 (54.11)		
Yes	1 (25.00)	3 (75.00)		
Pacemaker implantation *				0.177
No	64 (44.14)	81 (55.86)		
Yes	4 (80.00)	1 (20.00)		
History of malignant tumors *				0.184
No	66 (46.81)	75 (53.19)		
Yes	2 (22.22)	7 (77.78)		
History of diabetes			0.003	0.957
No	42 (45.16)	51 (54.84)		
Yes	26 (45.61)	31 (54.39)		
Admission pathways			2.541	0.111
Outpatient	55 (49.11)	57 (50.89)		
Emergency department and others	13 (34.21)	25 (65.79)		
Age ^#^	66.00 (61.00–72.00)	64.00 (57.00–75.00)	0.297	0.767
Gender			0.001	0.983
Female	14 (45.16)	17 (54.84)		
Male	54 (45.38)	65 (54.62)		
BMI ^##^	24.55 ± 3.14	23.71 ± 3.22	1.610	0.111
Smoking			0.003	0.955
No	31 (45.59)	37 (54.41)		
Yes	37 (45.12)	45 (54.88)		
Sleep duration ≥ 7 h/d			10.626	0.001
No	33 (35.11)	61 (64.89)		
Yes	35 (62.50)	21 (37.50)		
Educational level			0.204	0.652
0	9 (40.91)	13 (59.09)		
1	59 (46.09)	69 (53.91)		
Number of stents implanted			1.041	0.308
0	27 (50.94)	26 (49.06)		
1	41 (42.27)	56 (57.73)		
Operation duration ^#^	80.50 (61.50–105.00)	85.50 (65.00–112.00)	−0.789	0.430
Second operation			2.544	0.111
No	63 (47.73)	69 (52.27)		
Yes	5 (27.78)	13 (72.22)		
Hypersensitivity to contrast medium			2.845	0.092
No	65 (47.45)	72 (52.55)		
Yes	3 (23.08)	10 (76.92)		
Return to ward MAP ^#^	96.17 (90.00–103.17)	96.00 (89.67–102.67)	0.206	0.837
Return to ward blood glucose ^#^	7.80 (6.80–10.00)	8.90 (6.90–11.40)	−1.805	0.071
History of major life events			2.223	0.136
No	26 (54.17)	22 (45.83)		
Yes	42 (41.18)	60 (58.82)		
Baseline GAD-7 scores ^#^	2.00 (0.00–3.00)	3.00 (2.00–3.00)	−4.063	0.000
GAD-7 scores after operation ^#^	3.00 (2.00–4.00)	4.50 (4.00–5.00)	−5.147	0.000
Baseline PHQ-9 scores ^#^	1.00 (0.00–2.00)	3.00 (2.00–3.00)	−6.369	0.000

* The *p* value was calculated by the chi-square test. ^#^ The *p* value was calculated by Mann–Whitney U tests. ^##^ The *p* value was calculated by independent sample *t* tests. Abbreviations: PCI, percutaneous coronary intervention; CABG, coronary artery bypass grafting.

**Table 3:** Logistic regression analysis of risk factors for depression in ACS patients after PCI.

Variable	*B*	*S. E*	*Wald x^2^*	*p*	*OR (95% CI)*
Intercept	−4.648	0.878	28.033	0.000	
History of major life events	1.023	0.464	4.868	0.027	2.783 (1.121–6.907)
GAD-7 scores after operation	0.492	0.127	15.026	0.000	1.635 (1.275–2.097)
Baseline PHQ-9 scores	1.170	0.227	26.619	0.000	3.221 (2.065–5.023)

## Data Availability

The datasets generated or analyzed during this study are available from the corresponding author on reasonable request.
